# Measles susceptibility in maternal-infant dyads—Bamako, Mali

**DOI:** 10.1016/j.vaccine.2022.01.012

**Published:** 2022-02-23

**Authors:** Meredith G. Dixon, Milagritos D. Tapia, Kathleen Wannemuehler, Richard Luce, Mark Papania, Samba Sow, Myron M. Levine, Marcela F. Pasetti

**Affiliations:** a Global Immunization Division, US Centers for Disease Control and Prevention, Atlanta, GA, USA; b Center for Vaccine Development, University of Maryland School of Medicine, Baltimore, MD, USA; c Department of Biostatistics & Medical Informatics, University of Wisconsin – Madison, WI, USA; d Expanded Program on Immunization, WHO Regional Office for Africa, Inter-Country Support Team for West Africa, Ouagadougou, Burkina Faso; e Center for Vaccine Development, Ministry of Health, Bamako, Mali

**Keywords:** Measles, Mother-infant, Maternal antibodies, Measles plaque reduction neutralization, Measles immunization

## Abstract

Measles is endemic in Africa; measles mortality is highest among infants. Infant measles antibody titer at birth is related to maternal immune status. Older mothers are likelier to have had measles infection, which provides higher antibody titers than vaccine-induced immunity. We investigated the relationship between maternal age and measles susceptibility in mother-infant pairs in Mali through six months of infancy.

We measured serum measles antibodies in 340 mother-infant pairs by plaque reduction neutralization test (PRNT) and calculated the proportion of mothers with protective titers (>120 mIU/mL) at delivery and the proportion of infants with protective titers at birth, and at three and six months of age. We explored associations between maternal age and measles antibodies in mothers and infants at the timepoints noted.

Ten percent of Malian newborns were susceptible to measles; by six months nearly all were. Maternal and infant antibody titers were highly correlated. At delivery, 11% of mothers and 10% of newborns were susceptible to measles. By three and six months, infant susceptibility increased to 72% and 98%, respectively. Infants born to younger mothers were most susceptible at birth and three months. Time to susceptibility was 6.6 weeks in infants born to mothers with measles titer >120–<430 mIU/mL versus 15.4 weeks when mothers had titers ≥430 mIU/mL.

Maternal and newborn seroprotective status were positively correlated. Improved strategies are needed to protect susceptible infants from measles infection and death. Increasing measles immunization coverage in vaccine eligible populations, including nonimmune reproductive-aged women and older children should be considered.

## Highlights

• Maternal antibodies correlated with infant seroprotective status at birth.
• Ten percent of Malian newborns were susceptible to measles at birth.
• Nearly all infants were measles susceptible by six months of age.
• Improved strategies are needed to protect susceptible infants from measles death.

## 1 Introduction

Measles, one of the most contagious human viral diseases, causes an acute systemic infection with fever and respiratory compromise, often leading to serious complications such as blindness, encephalitis, severe pneumonia, and death [1]. In 2019, the World Health Organization (WHO) African (AFR) region reported 618,595 measles cases and an estimated 147,900 deaths, the highest figures among the six WHO regions [2]. Measles cases and deaths occur despite the existence of a safe, effective vaccine because of suboptimal immunization program performance [1]. Infants, many not yet eligible for vaccination, are at greatest risk of death [1], [3]. Recent reports of measles outbreaks and a measles surveillance analysis revealed a higher infection rate for infants than for other age groups [4], [5], [6], [7], which highlights the vulnerability of this group.

Before infants reach the age when they can receive measles vaccine, they rely on maternal antibodies primarily acquired transplacentally for protection against measles; when titers are measured to be >120 mIU/mL, an individual is considered protected [1], [8], [9]. It is generally believed that most infants are protected by maternal antibodies until six to nine months of age [1]. Level and duration of protection provided by maternal measles antibodies is influenced by maternal and infant factors [10]. Specifically, infection with wild-type measles virus induces higher measles titers compared with vaccination. Therefore, infants born to mothers who had been infected with wild-type measles virus have higher levels of maternally derived measles antibodies at birth and their protection is expected to last longer than infants born to vaccinated mothers [11], [12], [13], [14], [15], [16], [17]. The duration of acquired maternal protection is particularly relevant in AFR, where the first dose of measles containing vaccine (MCV1) in the routine immunization (RI) program is given to infants at nine months [1]; age at MCV1 administration is a program decision based on the level of measles transmission within the region. Countries and regions with low levels of measles transmission may administer MCV1 at 12 months to leverage the higher seroconversion rates that older age affords [1], [18].

In Mali, measles vaccination was first implemented alongside smallpox vaccination campaigns from 1967 to 1970 [19]. From 1980 to 1984, Mali reported a median of 8,759 cases annually [20], although these figures likely reflect underreporting. In 1985, Mali experienced a nationwide measles outbreak and reported 29,732 measles cases. That same year, MCV1 was introduced into the RI schedule, targeting children aged nine months of age [21], [22]. Additionally, supplemental immunization activities (SIA) were conducted in Mali to provide a second opportunity for MCV [23]. Despite MCV provision through RI and SIAs, persistent measles transmission continues to be reported, including among infants too young to be vaccinated [24], [25], [26].

A prospective study was conducted in Bamako, Mali, to examine the safety, immunogenicity, and efficacy of maternal influenza immunization during pregnancy for protection of infants against laboratory-confirmed influenza. The study involved active monitoring for influenza-like illness through the first six months of life. Serum samples were obtained longitudinally from mothers and infants at birth, and at three and six months that were consented for future use [27]. With access to stored specimens from mother-infant pairs obtained in the Mali influenza study, we investigated risk factors for infant susceptibility to measles at birth through six months of age, specifically maternal age and measles antibody level in mothers at delivery.

## 2 Methods

### 2.1 Maternal influenza study

For the original influenza vaccination trial, 4,425 mothers were recruited in the third trimester of pregnancy from referral and community health centers in urban Bamako, Mali, between September 2011 and April 2013; 4,193 of them were randomly assigned (1:1) to study intervention (trivalent inactivated influenza or quadrivalent meningococcal vaccine). Exclusion criteria, enrollment, and additional study methods have been described in detail elsewhere [27]. This study, including the measles antibody analysis in stored specimens, was approved by the Institutional Review Board of the University of Maryland School of Medicine; the ethics committee of the Faculté de Médecine, Pharmacie et Odonto-Stomatologie of Mali; the Ministry of Health of Mali; and the US Centers for Disease Control and Prevention. Mothers provided informed consent to blood draw for themselves and for their infants and for specimen storage and future use. Serum samples obtained at the local health care centers were transported to the Center for Vaccine Development in Bamako, Mali, then shipped to University of Maryland, Baltimore, USA, and stored at −80 °C. Information on study population for the parent influenza study had been collected via hand completed scannable forms (TeleForm, version 10.5.1; Autonomy Cooperation PLC) and populated a Microsoft Access database.

### 2.2 Study design and sample selection

A subsample of eligible mother-infant pairs (n = 340) was randomly selected from the maternal influenza study to investigate seroprotection status (circulating measles antibody levels) as a risk factor for infant susceptibility to measles. Mother-infant pairs were eligible for measles antibody analysis if the mother was 15–39 years old at the time of (singleton) birth and had completed all three scheduled visits at birth, three months, and six months.

### 2.3 Sample size

Mothers in the original study were stratified into five age groups: 15–19, 20–24, 25–29, 30–34, and 35–39 years. An equal number of mother-infant pairs (n = 68) in each stratum achieved 90% power to detect a linear trend in the proportion of infants with protective maternal measles titer across the five age groups using a Cochran-Armitage test and a significance level of 0.05. The sample size was estimated using PASS v 14.09 (NCSS, Kaysville, Utah, USA) assuming equally spaced proportions from 0.70 to 0.90.

### 2.4 Laboratory testing

Measles plaque reduction neutralization tests (PRNT) were performed, as previously described, with modifications [28]. Heat inactivated (30 min at 56 °C), serially diluted serum samples were incubated with an equal volume containing 30 plaque-forming units (pfu) of measles virus (Edmonston strain, ATCC, Manassas, VA) for 1.5 h at 37 °C, 5% CO_2_. The serum-virus mix was transferred onto ∼90% confluent Vero cell monolayers (ATCC) seeded in 24-well plates (in duplicate) and incubated for 1 h. After incubation the liquid was removed; cells were overlaid with 1% carboxymethylcellulose (1 mL, Sigma-Aldrich, St. Louis, MO) and incubated for 5 days at 37 °C, 5% CO_2_. On day 5, 0.5 mL of neutral red stain (Sigma-Aldrich, St. Louis, MO) diluted in Temin’s Modified Eagles Medium supplemented with 4% fetal bovine serum (Thermo Scientific, Waltham, MA) was added and incubated overnight at 37 °C, 5% CO_2_. The stained overlay was aspirated, fixed with 0.5 mL of 10% formalin solution (Millipore, Darmstadt, Germany) and allowed to dry overnight (covered). Each well was scanned using the Immunospot S6 Macro Analyzer. Cytopathic plaques were counted with Biospot Pro version 7.0 software (Cellular Technology Limited, Cleveland, OH). PRN titers were calculated using the Reed-Muench method as the inverse of the dilution that inhibited 50% plaques compared with no serum control and reported in mIU/mL based on the 3rd International WHO (97/648) reference standard (NIBSC, Hertfordshire, UK).

### 2.5 Data analysis

Date of birth or enrollment age was used to calculate maternal year of birth. Measles cases and MCV delivered via RI and SIA in Mali by year were plotted against maternal year of birth as a proxy for measles exposures [20]. PRNT > 120 mIU/mL were considered protective [29]. Maternal measles immune status was further classified into three categories, based on median titer at delivery: seronegative (≤120 mIU/mL), moderately positive (>120–430 mIU/mL), and strongly positive (>430 mIU/ mL). The Cochran-Armitage test for trend assessed whether the proportion of infants protected against measles at birth increased linearly with mother’s age. The Kruskal-Wallis test tested for differences in antibody titers among the five maternal age groups at birth (mothers and infants), three months, and six months. A loess smoother with span value = 0.7 was used to graph the relationship between maternal and infant titers. In an exploratory analysis, a linear mixed effect model was fit to the data to estimate the waning of infant measles titer over time. The model included a random intercept, time (weeks), the maternal measles immune status (seronegative, moderately positive, strongly positive), and the interaction between time and strength of response. The population average time, in weeks, to susceptibility was calculated from the estimated average decay of infants of mothers with moderately positive and strongly positive immune status. Analyses were conducted in SAS (version 9; SAS Institute) and R software (version 4.0.2; R Foundation); p-values ≤0.05 were considered statistically significant.

## 3 Results

**Fig. 1:**
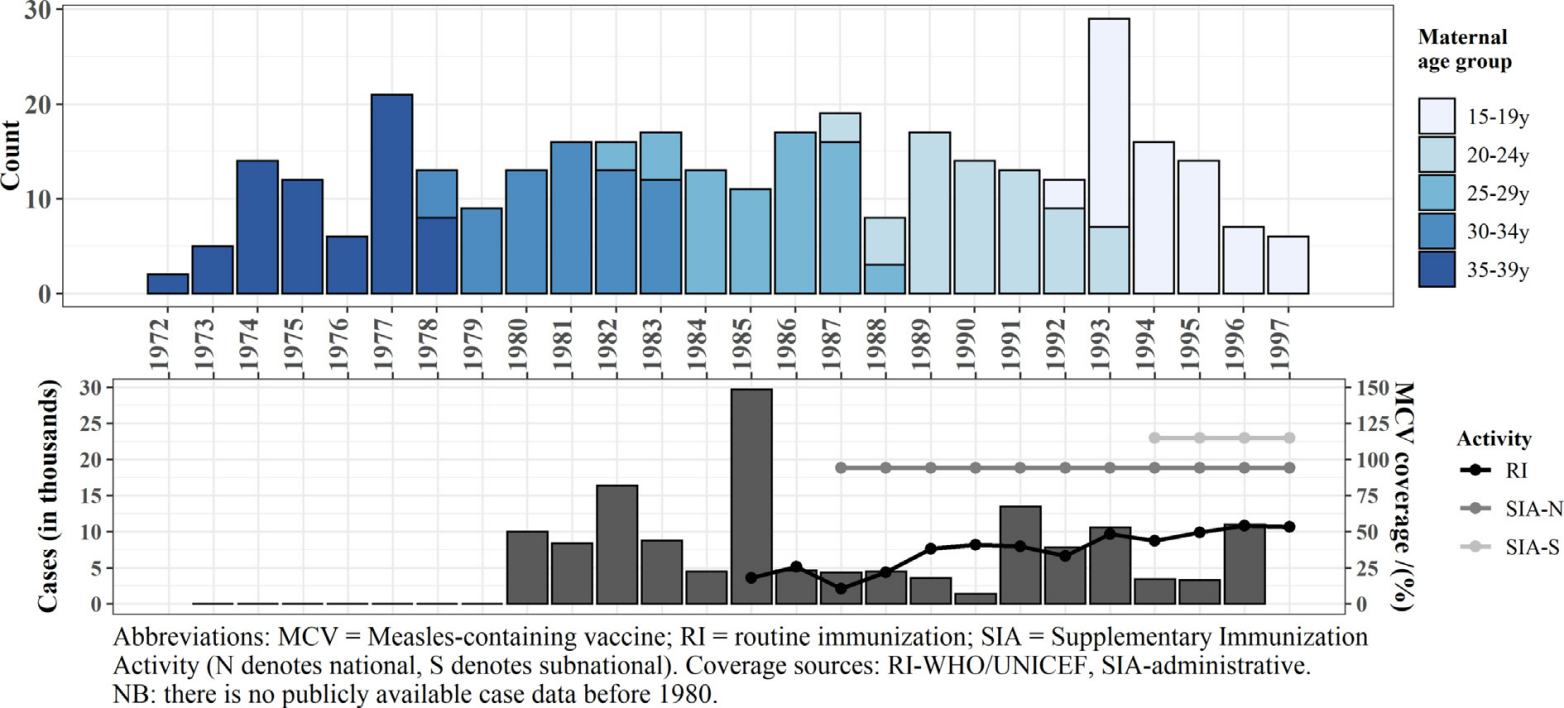
Number of study mothers by year of birth and age group—Bamako, Mali (top), and number of reported measles cases and vaccination coverage estimates from routine immunization (RI) and supplemental immunization activities (SIA)—Mali (bottom).

Maternal year of birth ranged from 1972 to 1997 (Fig. 1). Mothers born between 1972 and 1983, corresponding to all mothers in the 35–39 and 30–34 year age groups, and those born between 1982 and 1984, including some in the 25–29 year age group, would not have had the opportunity to receive MCV1 via RI (157; 46%). Those born in or after 1985, corresponding to most mothers in the 25–29 year age group and all in the 20–24 and 15–19 year age groups, may have had at least one opportunity for measles vaccination (183; 54%). Mothers born ≥1987, corresponding to 25–29, 20–24, and 15–19 year age groups, may have had two opportunities for measles vaccination (155; 46%). From the 340 selected mother-infant pairs, PRNT were missing from six mothers and for at least one time point from 38 infants due to insufficient volume or unavailable sample, including from one complete mother-infant pair.

Of mothers, an average of 11% lacked protective measles titers at delivery; percentages ranged from 6% in mothers in the 30–34 year age group up to 19% in mothers in the 25–29 year age group. The proportion of mothers susceptible at delivery was not associated with maternal age group (Cochran-Armitage test for trend, p-value = 0.31), although the 30–34 and 35–39 year age groups had higher measles antibody levels than mothers in younger age groups (Kruskal-Wallis p-value = 0.014) (Table 1).

**Table 1:** Summary of measles susceptibility rates and measles antibody titer measured by plaque reduction neutralization test (PRNT) in mothers at delivery and in infants at birth, and three and six months stratified by maternal age—Bamako, Mali. A titer ≤120 mIU/mL was considered susceptible. The Cochran-Armitage test for trend was used to test whether the proportion of infants protected against measles at birth increased linearly with mother’s age. The Kruskal-Wallis test was used to detect differences in the distributions of maternal measles titer at delivery and of infant’s measles titer at birth, and at three and six months among the five maternal age groups.

	Maternal age group	N	Number susceptible	Percent susceptible	Cochran-Armitage p-value	Median titer (mIU/ml)	25th percentile titer (mIU/ml)	75th percentile titer (mIU/ml)	Kruskal-Wallis p-value
**Maternal-delivery**	**Overall**	**334**	**37**	**11**	**0.31**	**432**	**212**	**957**	**0.014**
	15–19y	65	7	11		399	186	704	
	20–24y	68	8	12		386	199	670	
	25–29y	68	13	19		332	200	650	
	30–34y	67	4	6		599	313	1173	
	35–39y	66	5	8		566	266	1466	

**Infant-birth**	**Overall**	**322**	**32**	**10**	**0.01**	**490**	**235**	**1089**	**0.012**
	15–19y	62	13	21		420	166	789	
	20–24y	67	5	7		438	226	811	
	25–29y	63	7	11		395	194	739	
	30–34y	65	2	3		664	391	1508	
	35–39y	65	5	8		617	280	1490	

**Infant-3 months**	**Overall**	**326**	**236**	**72**	**0.01**	**56**	**29**	**130**	**0.024**
	15–19y	63	51	81		48	23	105	
	20–24y	68	53	78		52	28	102	
	25–29y	66	51	77		46	26	112	
	30–34y	66	38	58		96	35	180	
	35–39y	63	43	68		73	32	213	

**Infant-6 months**	**Overall**	**328**	**320**	**98**	**0.46**	**7**	**7**	**22**	**0.223**
	15–19y	65	63	97		7	7	19	
	20–24y	67	65	97		7	7	16	
	25–29y	66	64	97		7	7	17	
	30–34y	67	66	99		7	7	27	
	35–39y	63	62	98		9	7	27	

**Fig. 2:**
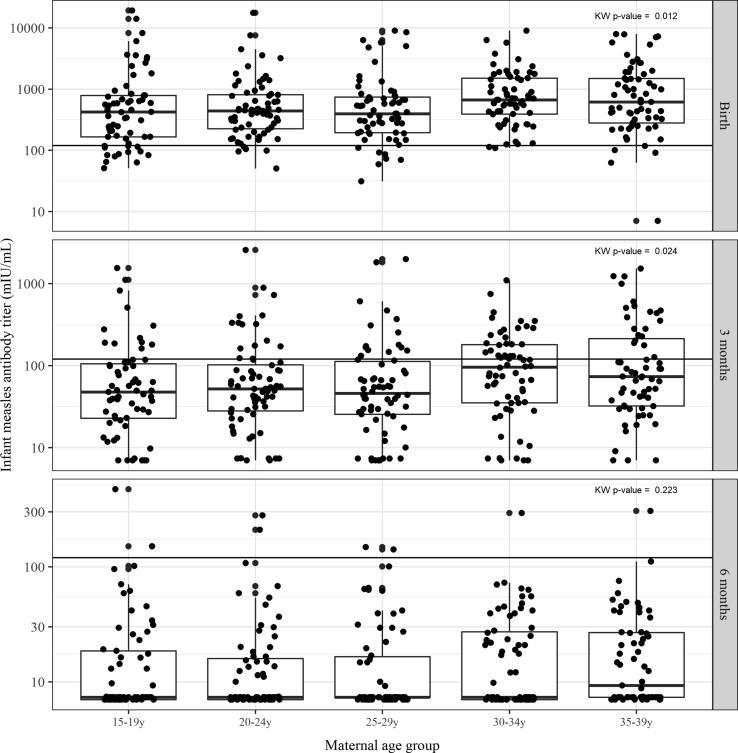
Boxplots depicting infant measles titer measured by plaque reduction neutralization test (PRNT) at birth stratified by maternal age group, on the log_10_ scale—Bamako, Mali. Dots represent each infant’s titer level. The 25th and 75th percentiles are represented by the bottom and top of the box, respectively. The dark line in the box’s interior represents the median. The black horizontal line across each graphic represents the threshold for protection (>120 mIU/ml). The Kruskal-Wallis test was used to detect differences in the distributions of maternal measles titer at delivery and of infant’s measles titer at birth, and at three and six months among the five maternal age groups.

**Fig. 3:**
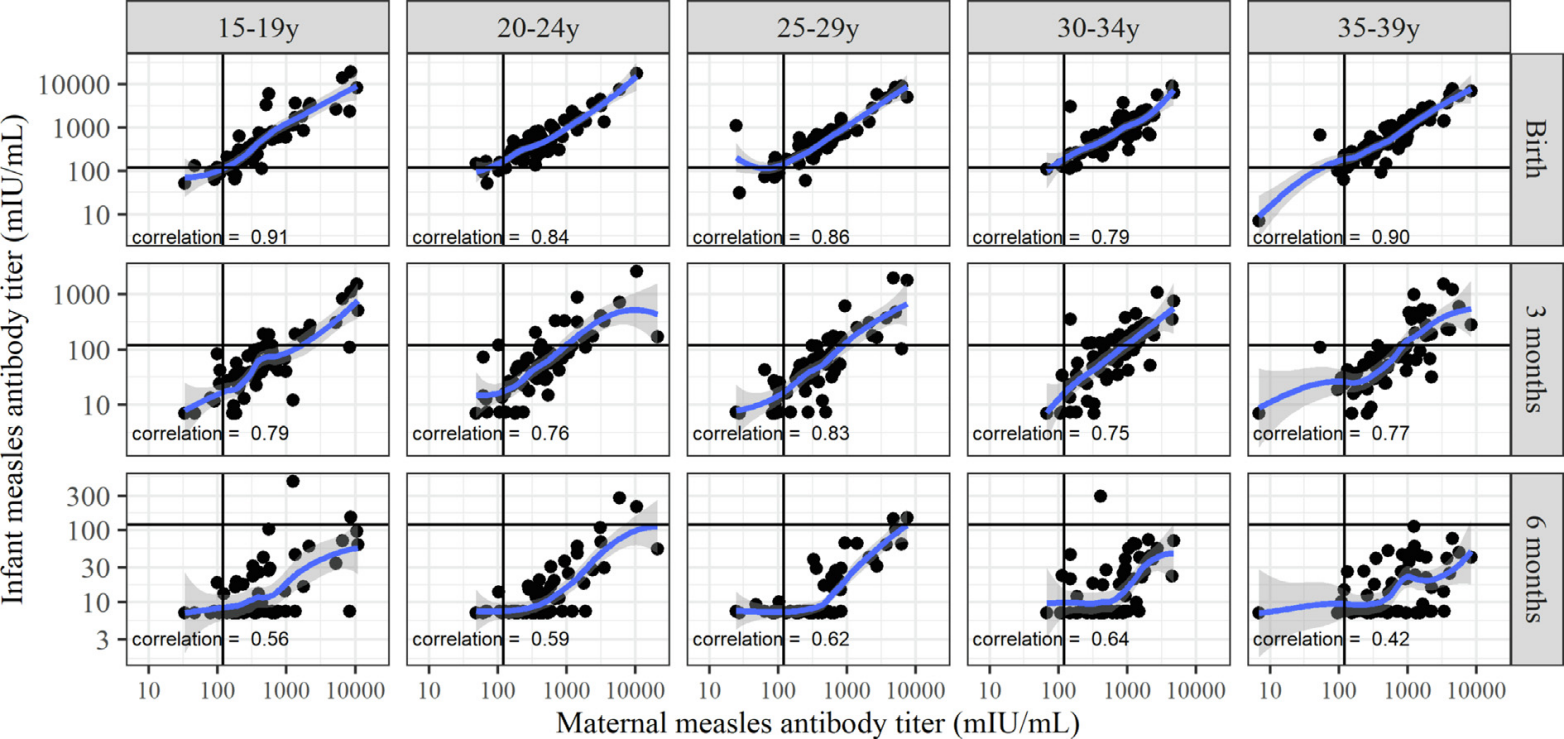
Dot plot of measles titer measured by plaque reduction neutralization test (PRNT) in mothers at delivery and in infants at birth, and at three and six months, stratified by maternal age group, on the log_10_ scale—Bamako, Mali. The blue spline represents the observed relationship between maternal and infant antibody levels. Black horizontal and vertical lines indicate the measles protective threshold (>120 mIU/ml) in mothers and infants. The estimated Spearman correlation coefficient is given in each panel.

Among infants, 10% were susceptible to measles at birth; the proportion of newborns susceptible to measles (<120 mIU/mL) was highest in those born to mothers aged 15–19 years (21%) and lower in those born to older maternal age groups (3–11%) (Cochran-Armitage test for trend, p-value = 0.01) (Table 1). Infants born to mothers in the 30–34 and 35–39 year age groups had higher titers at birth compared with infants born to younger mothers (Table 1 and Fig. 2: Kruskal-Wallis p-value = 0.012). There was a strong correlation between maternal measles antibodies at delivery and antibodies in infants at birth across all five maternal age groups, with Spearman correlation coefficients between 0.79 and 0.91 (Fig. 3). Mean infant: maternal measles antibody ratios at birth were 1.36 for mothers aged 15–19 years, 1.27 for mothers aged 20–24 years, 1.88 for mothers aged 25–29 years, 1.53 for mothers aged 30–34 years, and 1.34 for mothers aged 35–39 years (data not shown).

In infants, maternally derived measles antibodies decayed rapidly over time. By three months of age, most (72%) infants were susceptible to measles (Table 1, Fig. 2); 77–81% of infants born to younger mothers (ages 15–29 years) were susceptible, compared with 58–68% of infants born to older mothers (ages 30–39 years) (Cochran-Armitage test for trend, p-value = 0.01). Infants born to older mothers also had higher titers. By six months of age, 98% of infants were susceptible to measles, with no difference in susceptibility or antibody titers by maternal age group (Table 1, Fig. 2). The correlation between maternal measles antibodies at delivery and antibodies in infants was strong at birth, less strong at three months, and much weaker at 6 months since most infants had no or low antibody levels at that time (Fig. 3).

**Fig. 4:**
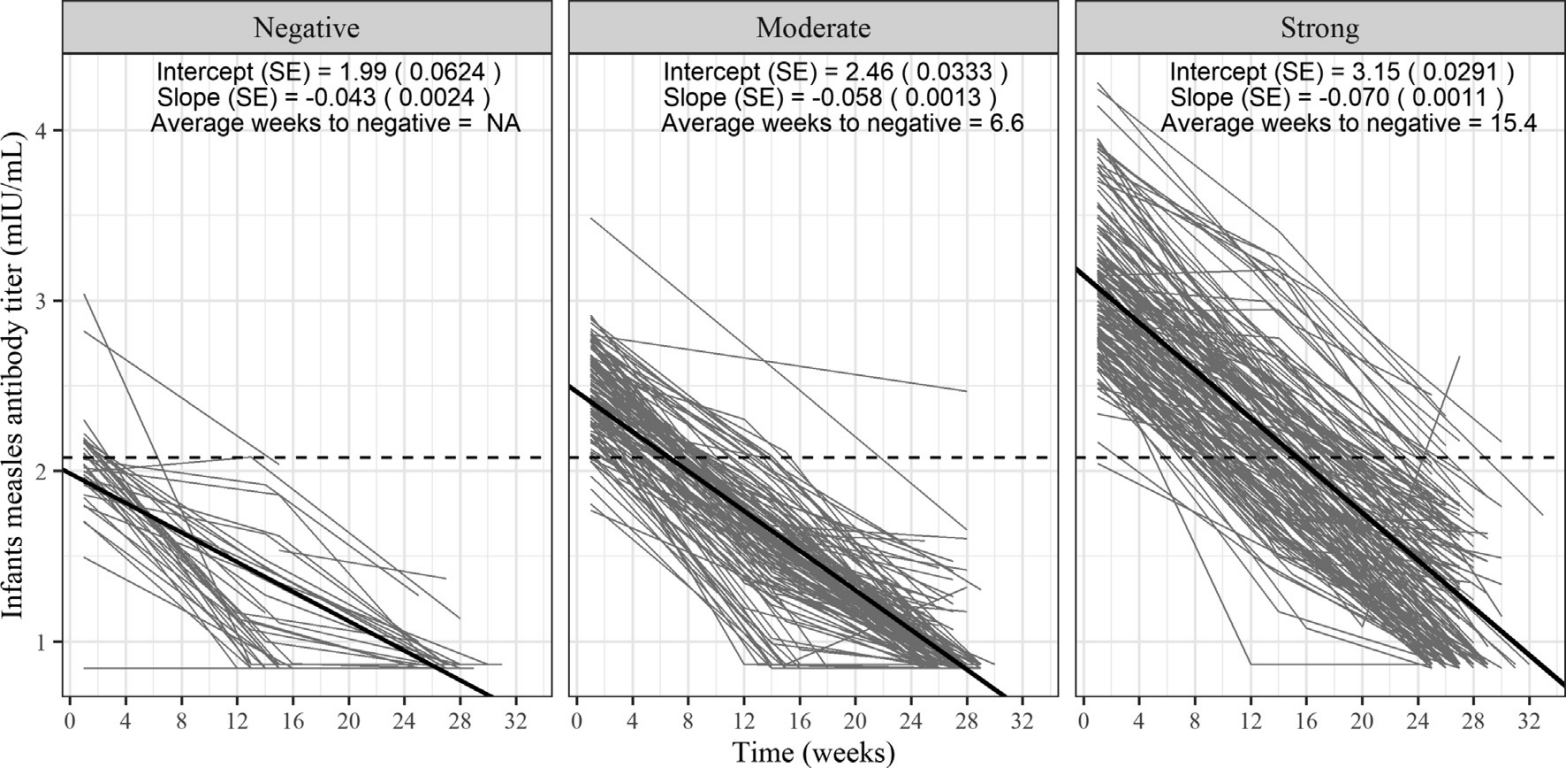
Decline of measles titers measured by plaque reduction neutralization test (PRNT) in infants born to mothers with different levels of measles immunity at the time of delivery: seronegative or unprotected (PRNT ≤ 120 mIU/mL), moderately positive (>120–<430 mIU/mL), and strongly positive (≥430 mIU/mL) in Bamako, Mali. Solid dark line is the estimated average decline from the random effects model; light gray lines represent each infant’s observed measles titer at birth, ∼3 months, and ∼6 months. The rate of measles antibody decline was marginally faster in infants born to mothers with a strongly positive immune status, as compared to those with a moderately positive status (F-test < 0.0001).

Estimates of average population and individual infant measles antibody decline in relation to maternal immune status are shown in Fig. 4. Infants of mothers who were not protected at delivery remained unprotected. The rate of measles antibody decline was marginally faster in infants born to mothers with a strongly positive immune status compared with those with a moderately positive status (F-test < 0.0001). Given the higher initial titers, the estimated time for maternally derived measles antibody titer to fall below the protective threshold was 15.4 weeks in infants whose mothers had strongly positive immune status compared with 6.6 weeks in infants whose mothers had moderately positive measles immune status.

## 4 Discussion

In this cohort of pregnant women recruited from health centers in Bamako, Mali, an area with endemic measles transmission and a measles vaccination program since 1985, we observed statistically significant positive associations between maternal age and infant measles antibody at delivery. Additionally, maternal age was associated with higher infant titers and protection against measles at three months. Measles titers in the infants at birth were highly correlated with maternal antibody titers, regardless of maternal age. Ten percent of infants were susceptible to measles at birth. By three months, most infants were susceptible to measles. At birth and three months, the proportion of susceptible infants was lower among those born to older mothers compared with infants born to younger ones, which could be explained by the higher measles titers among older mothers at the time of the study. By six months, virtually all infants were susceptible to measles, regardless of maternal age. Among the infants who were seroprotected the longest, most started with higher measles titers. Maternal measles antibody level at delivery was observed to have the strongest correlation with infant seroprotective status: infants born to mothers with a strongly positive immune status (>430 mIU/mL) had the highest average titers at birth and those antibodies lasted longer.

Higher measles titers in older mothers could be explained by increased likelihood that those born before 1985 had been infected by wild-type measles virus, while younger mothers may have received measles vaccine after its introduction into the RI program in 1985 and/or through measles SIAs. Also, as measles transmission decreased, reflected in fewer reported measles cases, opportunities for immunological boosting from exposure to circulating measles virus may have been reduced.

Some mothers lacked protective levels of measles antibodies and were susceptible to measles, which suggests that they were neither infected with wild-type measles virus nor effectively vaccinated. Less commonly, adult susceptibility may result from waning immunity. The infants born to susceptible mothers were susceptible to measles from birth. This is a serious risk in a region of high measles transmission because infants experience the most severe disease. Likewise, the observation that most three-month-old and virtually all six-month-old infants were susceptible to measles in this sample cohort is concerning. In this study population, measles susceptibility starts much earlier than anticipated, with susceptibility spanning >6 months before MCV1 eligibility for most infants.

In a previous study by Tapia, with a different sample of Malian infants, measles antibody levels were examined in infants with neither history of infection nor vaccination [30]. That study showed that 30% and 15% of two- and four-month-old infants had a protective measles titer, respectively, while none were protected at six months of age. A caveat with their analysis is that 200 mIU/mL was used as protective threshold; had >120 mIU/mL been applied, higher percentages of infants would have been considered protected. MCV1 coverage in Mali at the time of their study (2003) was 57% as compared to 56% in the year of birth for this study’s youngest mothers (1997).

To reduce infant vulnerability to measles, some authors have proposed decreasing the age of MCV1 administration [32]. However, MCV1 vaccine effectiveness varies by age at administration: 61% at 6–8 months, 84% at 9–11 months and 93% at ≥12 months [33], [34]. While providing a dose of MCV at six months of age may provide an individual infant with immunity against measles immediately, the corollary is reduced immunity at the population level. In the long term, such an approach, absent additional MCV doses, could result in even lower immunity among childbearing age women, who would transfer less measles antibody to their infants, subsequently leading to an even higher proportion of infants vulnerable to measles. MCV vaccination coverage in those ≥9 months of age needs to rapidly increase to fill immunity gaps, without an age cap for those who missed MCV1, to reduce measles virus transmission at the population level, and thus reduce the risk of infant infection, complications and death.

An important strength of our study is its prospective, longitudinal design. A second strength is the quantification of measles neutralizing antibodies. There is an accepted immune correlate against measles based on PRNT, considered the gold standard to predict protection from measles infection and disease [29], [35]. PRNT more sensitively detects measles antibodies than enzyme-linked immunosorbent assays. And PRNT results are directly comparable across laboratories because of a calibrated serum standard [36]. Another strength is the study context. Mali, a low-income, sub-Saharan African country [37], continues to have measles virus circulation. MCV1 coverage there remains low. MCV1 coverage in Mali in 2019 was 70%, a level well below the 95% coverage level with two timely doses of MCV required to achieve population immunity [31].

A second dose of measles-containing vaccine (MCV2), scheduled at age 12 months, was introduced in the RI program in December 2019. Our study population exhibits a wide seroprevalence and includes individuals who may have been previously infected with wild-type measles, vaccinated, both, or neither. As this region remains heavily affected by measles [2], and because the sub-Sahara African population is projected to double by 2050 [38], these findings are particularly relevant to inform future regional measles elimination efforts.

One study limitation is the lack of information on individual maternal and infant measles infection and vaccination, each of which may have influenced measles antibody titers. While we attempted to use maternal year of birth alongside population-level vaccination (RI, SIA) and case data as a proxy for measles exposures, these sources are not representative of individuals, and incidence of measles may be underreported due to weak surveillance [39]. A second limitation is that the sample is not representative of the wider Malian population, since pregnant women were recruited among those who attended health centers in the capital city of Bamako. Such mothers may have had an increased likelihood of both infection (due to more crowded urban living) and vaccination (due to easier programmatic access in a resourced location), thus limiting our ability to generalize this study’s findings to other regions.

## 5 Conclusions

In Bamako, Mali, infant measles titer at birth was highly correlated with maternal measles titer at delivery, regardless of maternal age. For both Malian mothers and infants, 10% or more were susceptible to measles at delivery and at birth, respectively. Infant susceptibility was higher among infants born to younger mothers. By three months, most infants were susceptible to measles and by six months virtually all infants were susceptible, leaving many infants susceptible for months before the recommended age of MCV1 administration. These results emphasize the urgent need to achieve high routine coverage at nine months of age in Mali and to increase measles immunization coverage among nonimmune reproductive-aged women and older children, including via catch-up vaccination throughout the life course. These steps would shield infants <9 months of age from measles infection, complications, and death. Lastly, more research is needed to understand measles susceptibility among reproductive-aged women and infants, particularly in the AFR region.

## Declaration of Competing Interest

The authors declare that they have no known competing financial interests or personal relationships that could have appeared to influence the work reported in this paper.
